# A nairovirus isolated from African bats causes haemorrhagic gastroenteritis and severe hepatic disease in mice

**DOI:** 10.1038/ncomms6651

**Published:** 2014-12-02

**Authors:** Akihiro Ishii, Keisuke Ueno, Yasuko Orba, Michihito Sasaki, Ladslav Moonga, Bernard M. Hang’ombe, Aaron S. Mweene, Takashi Umemura, Kimihito Ito, William W. Hall, Hirofumi Sawa

**Affiliations:** 1 Hokudai Center for Zoonosis Control in Zambia, Research Center for Zoonosis Control, Hokkaido University, N20, W10, Kita-ku, Sapporo 001-0020, Japan; 2 Department of Disease Control, School of Veterinary Medicine, University of Zambia, Great East Road, PO Box 32379, Lusaka 10101, Zambia; 3 Division of Bioinformatics, Research Center for Zoonosis Control, Hokkaido University, Sapporo 001-0020, Japan; 4 Division of Molecular Pathobiology, Research Center for Zoonosis Control, Hokkaido University, Sapporo 001-0020, Japan; 5 Department of Para-clinical studies, School of Veterinary Medicine, University of Zambia, Great East Road, PO Box 32379, Lusaka 10101, Zambia; 6 Department of Comparative Pathology, Graduate School of Veterinary Medicine, Hokkaido University, Sapporo 001-0020, Japan; 7 Centre for Research in Infectious Diseases, University College Dublin, Dublin 4, Ireland; 8 Global Virus Network, Baltimore, Maryland 21201, USA; 9 Global Institution for Collaborative Research and Education (GI-CoRE), Hokkaido University, Sapporo 001-0020, Japan

## Abstract

Bats can carry important zoonotic pathogens. Here we use a combination of next-generation sequencing and classical virus isolation methods to identify novel nairoviruses from bats captured from a cave in Zambia. This nairovirus infection is highly prevalent among giant leaf-nosed bats, *Hipposideros gigas* (detected in samples from 16 individuals out of 38). Whole-genome analysis of three viral isolates (11SB17, 11SB19 and 11SB23) reveals a typical bunyavirus tri-segmented genome. The strains form a single phylogenetic clade that is divergent from other known nairoviruses, and are hereafter designated as Leopards Hill virus (LPHV). When i.p. injected into mice, the 11SB17 strain causes only slight body weight loss, whereas 11SB23 produces acute and lethal disease closely resembling that observed with Crimean–Congo Haemorrhagic Fever virus in humans. We believe that our LPHV mouse model will be useful for research on the pathogenesis of nairoviral haemorrhagic disease.

## Abstract (web summary)

Bats carry viruses that can cause disease in other animals and in humans. Here, Ishii *et al.* identify new nairoviruses from African bats and show that some of them can produce a severe haemorrhagic disease in laboratory mice that is similar to Crimean–Congo haemorrhagic fever in humans.

## Graphical abstract

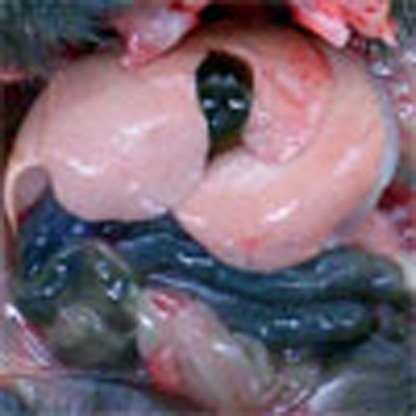

Bats have been shown to be important carriers of zoonotic pathogens^1^,^2^,^3^,^4^,^5^,^6^,^7^,^8^,^9^,^10^,^11^. In the last decade the introduction of high throughput next-generation sequencing (NGS) and metagenomic approaches have greatly facilitated the identification of a number of viruses in bats. Specifically this has allowed the detection of not only known, but also previously unrecognized viruses in a range of bat species from different geographical regions of both the Old^2^,^4^,^5^,^6^,^7^,^8^,^9^,^10^,^11^ and the New World^3^,^4^.

Severe zoonotic infectious diseases including fatal haemorrhagic fevers are important public health problems in Africa. The straw-coloured fruit bat (*Eidolon helvum*) is involved in the circulation of potentially zoonotic henipaviruses and Egyptian fruit bat (*Rousettus aegyptiacus*) is a likely reservoir of Marburg virus (MARV) and viruses in the genus *Ebolavirus*, which cause haemorrhagic fevers in Africa^2^,^8^. Other haemorrhagic fever-related viruses in Africa are hosted by rodents and ticks. Lassa virus in the family Arenaviridae is hosted by a rodent of *Mastomys natalensis*, however, the host of Lujo virus is still unknown^12^,^13^,^14^,^15^,^16^. Crimean–Congo Haemorrhagic Fever virus (CCHFV), is a member of the family *Bunyaviridae* and causes CCHF by transmission via Ixodid ticks from rodents and/or other animal species^17^,^18^,^19^.

In the present study, we focus on the identification of novel viruses in bats, which are associated with haemorrhagic fever, and development of small animal models for such agents. Specifically we have employed a combination of NGS approaches with classical virus isolation methods employing cell cultures and *in vivo* studies to investigate clinical and pathological features of disease. Herein we describe the isolation of novel nairoviruses from *Hipposideros gigas* bats in Zambia and demonstrate that specific strains of this virus produce a profound haemorrhagic disease in mice, which is clinically and pathologically similar to human CCHF.

## Results

### Discovery of Leopards Hill virus

A total of 59 bats were collected in the Leopards Hill cave in Lusaka, Zambia (15° 36.132′ S, 28° 43.457′ E) between 2010 and 2012. Harvested tissues from bats were homogenized and inoculated onto Vero E6 cells. In initial studies, culture supernatants were collected following 14 days of incubation. RNAs were extracted from supernatant samples and complementary DNAs (cDNAs) were prepared. Random-shotgun sequencing using NGS demonstrated the presence of nairovirus-related genome sequences in the supernatants (Supplementary Fig. 1). The results showed that 16,626 of 30,252 and 30,894 of 50,583 reads from the culture supernatants inoculated with liver and lung homogenates from bats collected in 2011, respectively, could be annotated to nairovirus sequences using BLAST searches.

One-step reverse transcription PCR (RT–PCR) was subsequently performed with primers based on the obtained nucleotide sequences to specifically amplify the nairovirus genome in each sample. The bat species involved were identified based on nucleotide sequences of the *cytochrome b* (*cytb*) gene and it could be shown that nairovirus-related RNAs were exclusively detected in bats of the species *H. gigas*, which had been sampled in 2010 and 2011, with prevalence of 77.8% (7 of 9) and 31.0% (9 of 29), respectively. Tentatively, this nairovirus was designated Leopards Hill Virus (LPHV). In this study, LPHV RNA was not detected in tissues from other captured bats. These were 1 *H. gigas* collected in 2012, 8 *Minipteros* sp., 5 *Myotis* sp., 3 *Rhinolophus* spp. and 4 *R. aegyptiacus*.

To begin to investigate genetic diversity within the LPHVs we carried out nucleotide sequencing and phylogenetic analysis of RT–PCR amplicons from the L segment of all 16 positive samples (Supplementary Fig. 2). Specifically 15 of the 16 had closely related nucleotide sequences (100 to 96.9% identity). However the remaining one sequence (11SB24) clustered distinctly. The mean identities at the nucleotide (nt) and amino acid sequence levels between 11SB24 and the other LPHVs were ~60% and 53.8%, respectively. These preliminary studies suggest that the LPHVs may be a genetically diverse group of viruses and possibly that there may be distinct genetic subtypes. Unfortunately the 11SB24 virus could not be isolated and it is unknown if this may have unique biological or pathogenic properties.

### Isolation of virus and whole-genome analysis

Homogenates from LPHV genome-positive liver and lung tissues were inoculated onto Vero E6 cells and cultured for 7 days. After a second passage in Vero E6 cells, viral RNAs (vRNAs) of two isolates from lung tissues (11SB17 and 11SB19) and an isolate from liver tissue (11SB23) were prepared from culture supernatants. Genomic sequences of these isolates were analyzed using NGS, and it could be confirmed that the genomic organization and sequences displayed characteristics typical of bunyaviruses with tripartite L, M and S genome segments. The nairovirus genome is a tripartite (L, M and S) negative sense single-stranded RNA. The L gene produces a single protein—the RNA-dependent RNA polymerase. The M segment encodes a glycoprotein precursor (GPC), which is processed into two structural envelope proteins: glycoprotein Gn and Gc. The S segment encodes the nucleocapsid N protein.

**Figure 1:**
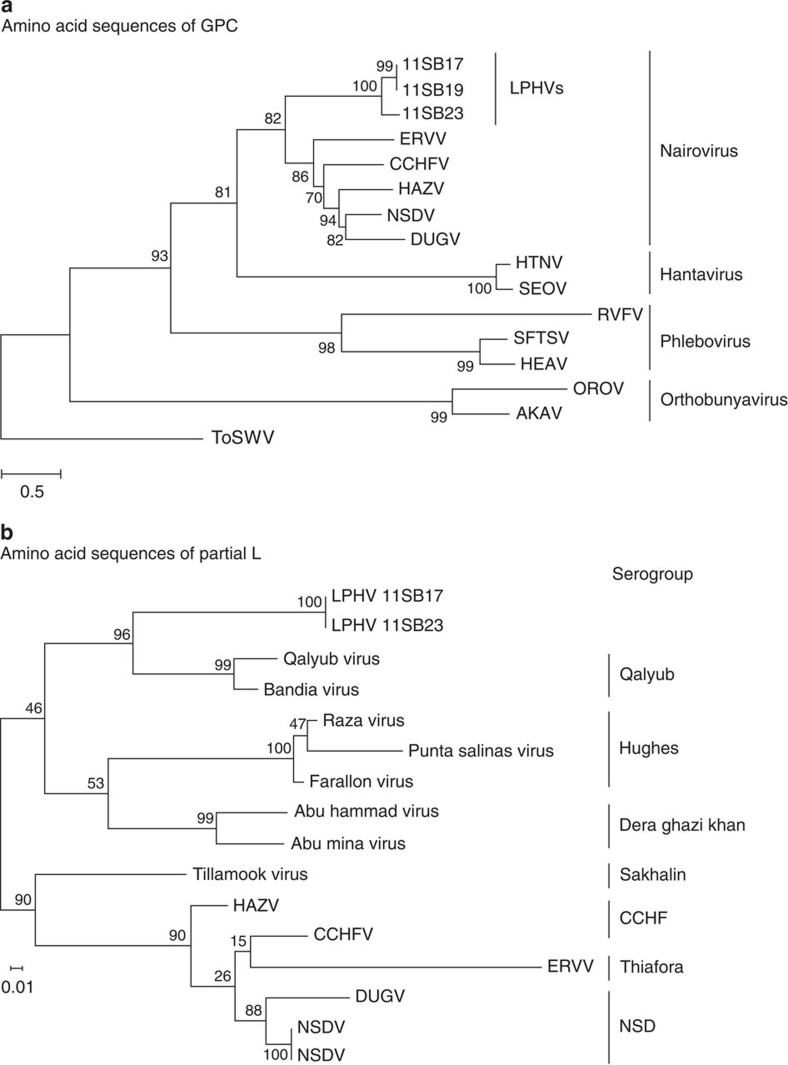
Phylogenetic analysis of LPHVs and other bunyaviruses based on amino acid sequences. The percentages of trees in which the associated taxa are clustered are shown beside the branches. The tree is drawn to scale, with branch lengths indicating the number of substitutions per site. The analysis involved 16 amino acid sequences. All positions containing gaps and missing data were eliminated. (**a**) Amino acid sequences of the GPC were phylogenetically analyzed, and the tree with the highest log-likelihood value (−31327.7075) is shown. There were a total of 933 positions in the final data set. (**b**) Amino acid sequences of the partial L protein were phylogenetically analyzed, and the tree with the highest log-likelihood value (−1582.7832) is shown. There were a total of 137 positions in the final data set. Nairoviruses were designated by the following IDs: AKAV, akabane virus; HEAV, heartland virus; HTNV, hantaan virus; OROV, oropouche virus; RVFV, rift valley fever virus; SEOV, Seoul virus; SFTSV, severe fever with thrombocytopenia syndrome; ToSWV, tomato spotted wilt virus. ToSWV was defined as the out group.

The complete genomic sequences of the 11SB17 and 11SB23 strains and partial genomic sequence of the 11SB19 strain were determined and this allowed characterization of complete sequences of the L, GPC and N genes (Supplementary Table 1). Phylogenetic analysis of the deduced amino acid sequences of the full-length GPC proteins from the three LPHV strains are shown in Fig. 1a. This clearly showed that the LPHVs belonged to genus *Nairovirus* and were highly divergent from other known nairoviruses. In addition, this analysis showed significant divergence in the sequences of the precursor glycoprotein GPC of the 11SB23 strain compared with that of 11SB17 and 11SB19. While the nucleotide and amino acid sequences of N and L were highly conserved among LPHVs (>99.0% and >99.6% identity at the nucleotide and amino acid levels, respectively), the GPC of the 11SB23 strain differed significantly from that of the 11SB17 and 11SB19 strains (identities were 68.1% and 73.1% at the nucleotide and amino acid sequence levels, respectively). On the basis of the amino acid sequence homologies, the 11SB19 and 11SB17 strains were considered identical.

Pairwise comparisons of nucleotide sequences of each gene and amino acid sequences of each protein among the isolated LPHVs were made with CCHFV, Erve virus (ERVV), Nairobi Sheep Disease Virus (NSDV) and Hantaan virus in the genus *Hantavirus* and these are shown in Table 1. The mean sequence identities of the N gene and protein for the LPHVs and other Nairoviruses were 42.2% at the nucleotide and 29.9% at the amino acid levels and that for HNTV was 26.5% and 10.1%, respectively. The mean sequence identities of the L gene and protein for the LPHVs and other Nairoviruses were 48.3% and 39.6% at the nucleotide and amino acid levels, respectively, and that for HNTV was 19.3% and 7.1%, respectively. The mean sequence identities of the GPC gene and protein for the LPHVs and other nairoviruses were 34.0% and 22.8% at the nucleotide and amino acid levels, respectively, and that for HNTV was 26.8% and 9.7%, respectively.

In the genus Nairovirus, several serogroups can be differentiated based on phylogenetic analysis of a partial sequence of the L gene^20^. This study also demonstrated that that the observed genetic differences in nairoviruses reflects the diversity of their predominant tick hosts and suggested that there could be co-evolution of the viruses and ticks^20^. We carried out a similar analysis of the LHPVs and found that while they formed a distinct phylogroup (Fig. 1b) they clustered closely with the Qalyub serogroup, which is known to be associated with soft tick genus *Ornithodoros.* However an examination of the all of the bats in our study failed to find evidence of tick infestation. Further studies are required to further investigate this and to determine what role ticks may have, if any, in the transmission of LPHVs.

It is known that the L protein of CCHFV and other nairoviruses contain a viral ovarian tumour (vOTU)-domain, which has been implicated as a virulence factor, and in the case of CCHFV this may be related to interference with innate immune responses^21^. We have also identified a vOTU in the LPHVs (Supplementary Fig. 3B).

There was over 99% identity between the 11SB17 and 11SB23 isolates with only one amino acid difference (R/K) being observed. Comparison of the amino acid sequences of the vOTU domains in the LHPVs and other nairoviruses are shown in Supplementary Fig. 3B and it could be seen that the LPHVs had greatest homology with that of Dugbe virus (DUGV) with a 37% identity at the amino acid level.

### LPHV M-gene segment and glycoprotein processing

The nairovirus glycoproteins encoded by the M segment are processed from a polyprotein by proteases, signalases, SKI-1/S1P, furin and convertases^22^,^23^,^24^. In CCHFV, the M gene encodes a single polyprotein, which is processed through co-translational cleavage and post-translational processing into two transmembrane structural glycoproteins Gn and Gc (ref. 19). The M segment of CCHFV contains two additional domains, the mucin and GP38 domains whose function is unclear. The M segment also encodes a non-structural protein NSm whose function is also unknown^22^.

**Figure 2:**
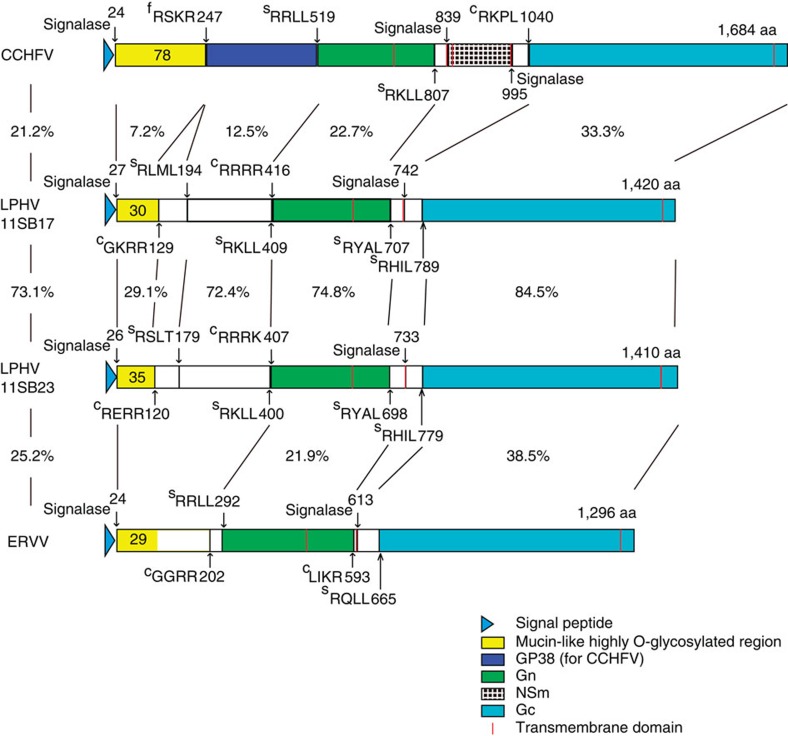
Comparison of deduced glycoprotein structures. Glycoprotein precursors of the two LPHV strains and ERVV were deduced on the basis of primary structures and known information for CCHFV. Identified and deduced proteolytic sites are indicated by positions of polyproteins after tetrapeptide sequences that represent protease recognition sites. Proteases that recognize each site are indicated at the left side of the tetrapeptide sequences: c, supposed precursor convertase as described in a previous study; f, furin and s, SKI-1/S1P ref. 42. Deduced cleavage site after endogenous signal peptide sequence by signalase are indicated. Predicted numbers of O-glycosylated residues are indicated in the mucin-like domain. The homologies of 11SB17 and 11SB23 domains are indicated. Percentage homologies of the full-length glycoprotein precursor and each domain are indicated between the strain names and the domains.

Predicted proteolytic sites in the LPHV polyprotein based on those reported for CCHFV and the predicted mature structures of the LPHV glycoproteins are schematically represented in Fig. 2. The highly O-glycosylated mucin domain in CCHFV is also present in the N terminus of the LPHV polyprotein. The region corresponding to the CCHFV GP38, which is also present and adjacent to the mucin domain, was present in the 11SB17 and 11SB23 strains and had 72.4% amino acid identity. However neither of these protein sequences could be annotated to known proteins in BLAST searches. The structural Gn and Gc proteins of CCHFV contain transmembrane domains in their C-termini. Similar structures were identified in the middle and in the C terminus of the LPHV polyproteins. The Gn and Gc from the two LPHVs had 74.8% and 84.5% amino acid identity, respectively. BLAST search analysis confirmed that the amino acid sequences of LPHV Gn and Gc peptides were similar to those of other nairoviruses (% identity was ~20%–22% and 29%–38%, respectively). The NSm protein in CCHFV is located between Gn and Gc and contains three transmembrane domains. This has also been identified in the polyproteins of other nairoviruses including NSDV, DUGV and Hazara virus (HAZV). In contrast, only one transmembrane domain was identified in the region between Gn and Gc in the two LPHVs and the predicted sequence of NSm was not evident. Notably another nairovirus, ERVV, which was isolated from a shrew^25^,^26^ also had only one transmembrane domain and NSm was also not found (Fig. 2) Thus LPHVs and ERVV are similar in that they lack the NSm domain between Gn and Gc.

### Clinical findings in mice inoculated with LPHVs

**Figure 3:**
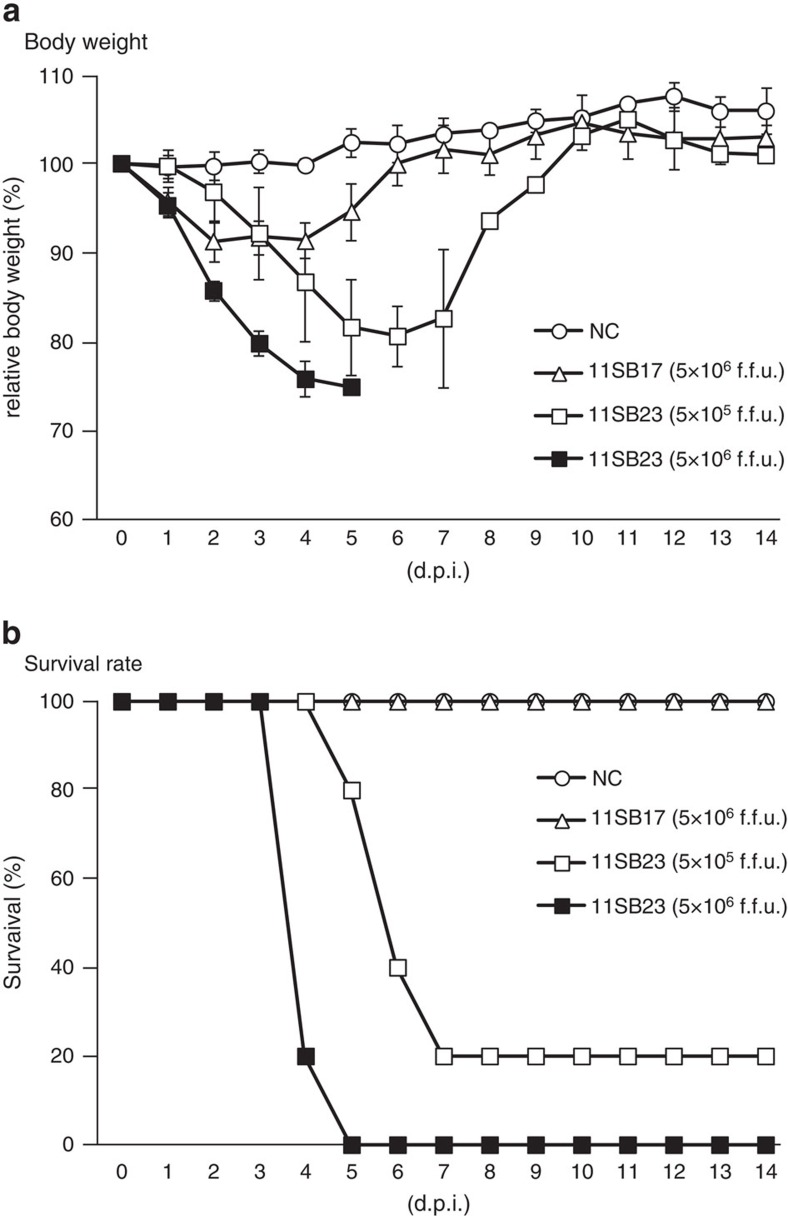
Clinical indicators of LPHV infection in C57BL/6 mice. 11SB17 and 11SB23 strains at the indicated titres or PBS (NC) were i.p. injected into C57BL/6 mice. Five individuals (*n*=5) were challenged for each condition. (**a**) Body weight changes in LPHV-infected mice were monitored. Average relative body weights are indicated with s.d. (**b**) Survival rates of mice after LPHV challenges or PBS injections are indicated.

To characterize the pathogenicity of LPHV, the 11SB17 and 11SB23 strains were i.p. injected into C57BL/6J mice. About 5 × 10^6^ fluorescent focus forming unit (f.f.u.) and 5 × 10^5^ f.f.u. of the 11SB23 strain in PBS were injected to represent high and low-dose challenges, respectively. The LPHV 11SB17 strain was inoculated only at 5 × 10^6^ f.f.u. PBS was used as a negative control (NC). Body weights of the challenged mice were monitored daily (Fig. 3a). From 2–7 days post infection (d.p.i.), mice injected with the 11SB23 strain showed significant body weight loss. High and low inocula of the 11SB23 strain caused 100% (5/5) and 80% (4/5) mortality by 7 d.p.i., respectively (Fig. 3b). Clinically, mice were observed to curl up, and exhibited shivering without movement before death. Infection with a high inocula of the 11SB17 strain resulted in only a slight body weight loss between 2–4 d.p.i. and all mice survived (Fig. 3b). At 21 d.p.i., serum antibodies in all surviving mice were examined using enzyme-linked immunosorbent assay (ELISA), and all were found to have high-titres of immunoglobulin-G (IgG) antibody to LPHV (Supplementary Fig. 4).

### Pathological findings in mice infected with LPHVs

**Figure 4:**
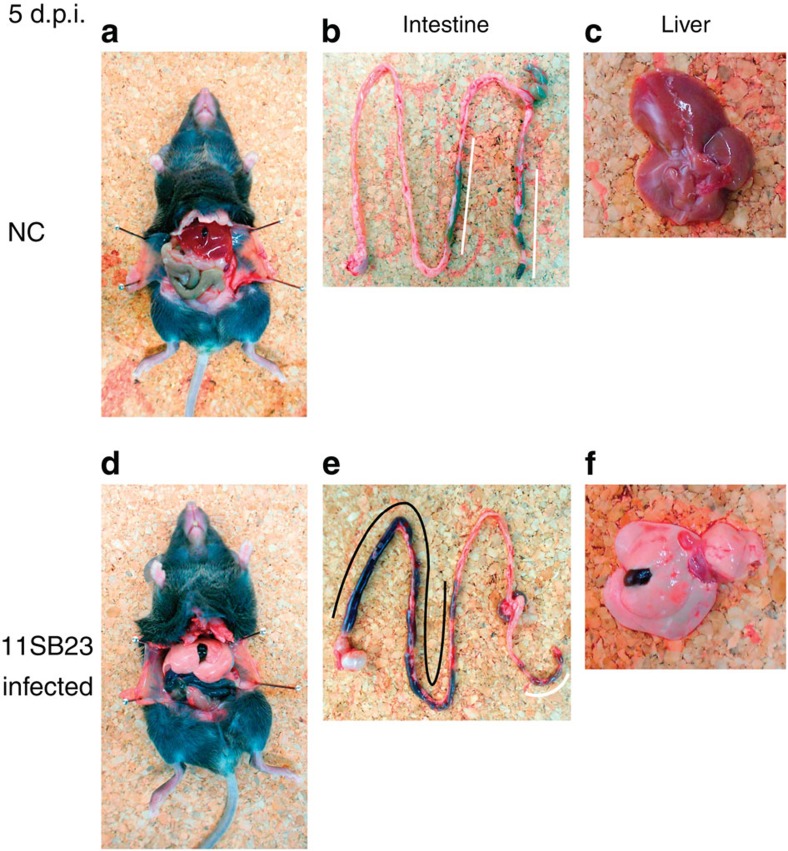
Macroscopic changes in LPHV-infected mice. C57BL/6 mice injected with PBS (NC; **a**–**c**) and high inoculum dose of the 11SB23 strain (**d**–**f**) were dissected at 5 d.p.i. Whole body, intestine and liver photographs are shown. Bleeding region in the small intestine of 11SB23-infected mouse is indicated by black line (**e**), and faeces in colon and rectum were indicated by white line.

LPHV-injected mice were examined and on gross examination livers from 11SB23-infected animals were found to be whitened compared with those from uninfected mice (Fig. 4a,c,d,f). The intestines were found to have black contents, suggestive of gastrointestinal bleeding (Fig. 4e). In contrast the colon and rectum of uninfected mice did not exhibit evidence of haemorrhage and only contained faeces (Fig. 4b). Hearts, lungs and kidneys from the infected mice did not show any remarkable pathological findings. Macroscopically, bleeding in the intestines was observed in all mice infected with the LPHV 11SB23 strain (data not shown). Notably no pathological changes were observed in mice infected with the 11SB17 isolate, which would be consistent with their mild clinical presentation and absence of overt illness. At 21 d.p.i., macroscopic examination of tissues from all mice, which had recovered, showed similar findings to those in uninfected mice (data not shown).

**Figure 5:**
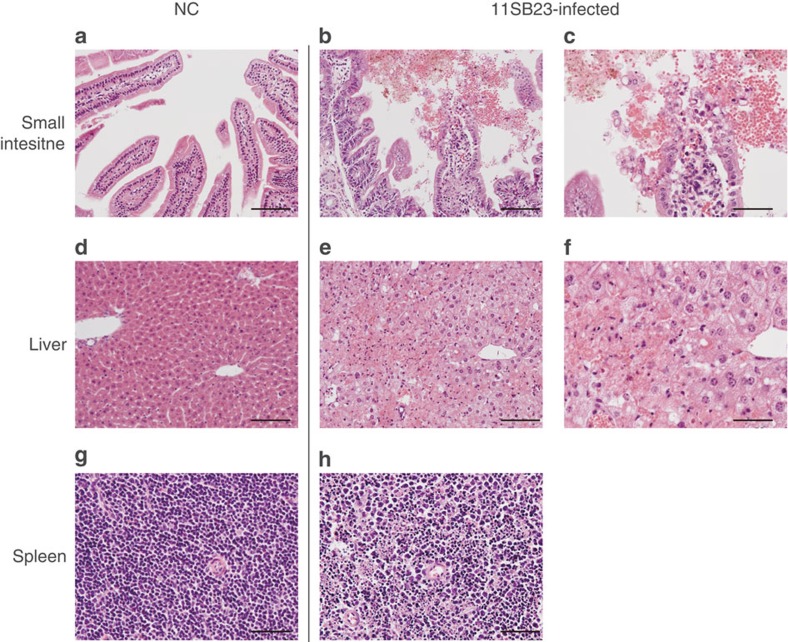
Histological findings of virus-infected tissues. C57BL/6 mice injected with PBS (NC; **a**,**d**,**g**) and high dose of 11SB23 (**b**,**c**,**e**,**f**,**h**) were dissected at 5 d.p.i. Small intestine, liver and spleen tissues are shown with haematoxylin–eosin staining after fixation in 10% formalin. Small intestine and liver were observed by × 20 (panels in the left and middle column) and × 40 (panels in the right column) objective lens and that for the spleen was × 20. Scale bars are shown in the lower right side of the figures (**a**,**b**,**d**,**e**: 100 μm; **c**,**f**,**g**,**h**: 50 μm).

Small intestine, liver and spleen in uninfected NCs exhibited no pathological changes (Fig. 5a,d,g). In contrast, the intestines of 11SB23-infected mice showed haemorrhage, hydropic degeneration and exfoliation of mucosal epithelial cells at the villous tip of the small intestine (Fig. 5b,c). The epithelium in the glandular stomach had similar pathological changes (data not shown). Severe and prominent hydropic swelling and necrosis of hepatocytes was found in all 11SB23-infected mice (Fig. 5e,f). Hepatocytes of these mice were invariably swollen and had pale granular cytoplasms compressing the hepatic sinusoids and there was evidence of single-cell focal necrosis.

**Figure 6:**
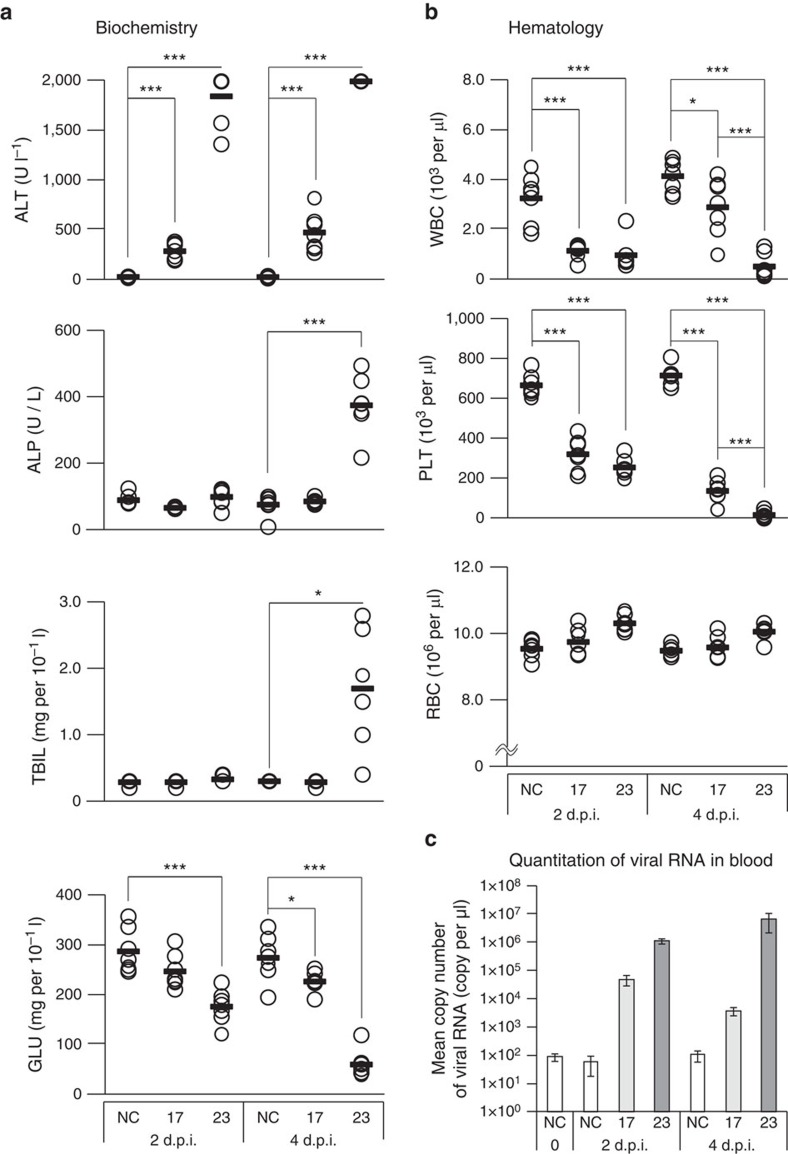
Blood tests and quantification of viremia in non-infected and LPHV-infected mice. C57BL/6J mice were i.p. injected with PBS (NC) or 5 × 10^6^ f.f.u. of LPHV 11SB17 (17) or 11SB23 (23) strain. At the indicated time points, peripheral blood was harvested from seven individual animals (*n*=7). Individual values (open circle) and their averages (solid bar) were indicated. ***P<0.001 and *P<0.05 (Welch’s *t*-test). (**a**) Values of alanine aminotransferase (ALT), alkaline phosphatase (ALP), total bilirubin (TBIL) and glucose (GLU) are indicated. At 4 d.p.i. in 11SB23-infected mice, a value of TBIL affected by haemolysis was removed from the analysis (*n*=6). The maximum measureable value of ALT was 2,000, so values beyond 2,000 were recorded as 2,000. (**b**) Number of white blood cells (WBC), platelets (PLT) and red blood cells (RBC) are indicated. (**c**) Mean copy numbers of viral RNA in the three representative individuals of peripheral blood were measured by qRT–PCR. In each infection, this was measured at 2 and 4 d.p.i. Negative control (NC) at 0 d.p.i. indicated non-specific background levels and each value was expressed as mean±s.d. *Y*-axis is a logarithmic scale.

Spleens of these mice also exhibited widespread necrosis of splenocytes (Fig. 5h). Blood biochemistry and haematology tests of LPHV-infected mice were carried out at 2 and 4 d.p.i. Biochemistry tests showed elevated levels of alanine aminotransferase (ALT), alkaline phosphatase, total bilirubin and decreased glucose concentrations in 11SB23-infected mice (Fig. 6a). A summary of the laboratory data is shown in Table 2. ALT levels in 11SB23-infected mice increased rapidly from 2 d.p.i. At 4 d.p.i., high levels of bilirubin and amylase were also observed and this was consistent with the progression of liver disease. ALT levels in 11SB17 infection were also elevated but at much lower levels compared with 11SB23 infection. Differences in ALT levels reflected the severity of liver injury. In this respect, gross pathological findings of liver in 11SB17 infection showed no obvious abnormalities either at 2 and 4 d.p.i. (data not shown). Alkaline phosphatase and total bilirubin levels in 11SB17 infection at both days remained close to normal values and the level of glucose concentration was found to be close to normal at 2 d.p.i. and only slightly decreased at 4 d.p.i.

Haematology testing revealed severe leucopenia and thrombocytopenia in both 11SB17- and 11SB23-infected mice (Fig. 6b). At 4 d.p.i., the counts of white blood cells and platelets in 11SB17-infected mice were much lower than NC mice but significantly higher than 11SB23-infected mice. Copy numbers of vRNA in peripheral blood were measured by quantitative RT–PCR (Fig. 6c), and these were significantly higher in 11SB23-infected mice. At 2 d.p.i., mean copy numbers of vRNA in the peripheral blood were ~4.8 × 10^4^ copy per μl and 1.1 × 10^6^ copy per μl in 11SB17 and 11SB23 infection, respectively. At 4 d.p.i., these were ~3.7 × 10^3^ copy per μl and 6.5 × 10^6^ copy per μl, respectively. In contrast, the value related to non-specific amplification in this assay was 1 × 10^2^ copy per μl. The relative levels of LPHV RNA in tissues were also determined using quantitative RT–PCR (qRT–PCR) at 21 d.p.i. or on the day of sacrifice (Supplementary Fig. 5). In lung, spleen and kidney, relative levels of vRNA in 11SB23-infected mice (5 and 6 in Supplementary Fig. 5) were ~500–1,000-fold higher than those in the 11SB17-infected mice.

To determine if the differences in the pathogenicity of the LPHVs could be related to possible differences in their growth or replication kinetics, we compared their replication rates in different cell lines including IFN-deficient Vero E6 cells, IFN competent HuH-7 cells and IFN competent Tb1Lu cells using qRT–PCR to measure the production of genomic RNA in infected culture supernatants at different times after infection. These studies demonstrated (Supplementary Fig. 3A) that the growth kinetics were very similar if not identical in all three cell lines.

## Discussion

Unknown pathogens which circulate in wild animals are sources of emerging infectious disease in humans, and in recent years bats have been shown to serve as important reservoirs of pathogens^1^,^2^,^3^,^6^,^7^,^8^,^9^,^10^,^11^. In relation to viruses known to be associated with the development of haemorrhagic fevers the most likely host of MARV is the fruit bat *R. aegyptiacus* in Africa and recently a novel ebolavirus-like filovirus has been found in *Miniopterus schreibersii* in Spain^2^,^27^. In the present study, we focused on the identification of viruses in bats collected in Zambia with the goal of identifying agents associated with haemorrhagic disease. In these studies we employed a combination of both modern molecular approaches, specifically NGS together with classical virus isolation methods and *in vivo* animal studies to characterize viruses in selected bat populations. Initial studies employing highly efficient random-shotgun sequencing revealed the presence of novel nairovirus genomic sequences in bats collected in Zambia between 2010 and 2012. This virus was named LPHV, and RT–PCR studies demonstrated that infection exclusively involved the *H. gigas* species of bat (16/38; 42%).

Phylogenetic analysis based on the nucleotide sequences of the L gene fragment showed that the LPHVs obtained were distinct from all known nairoviruses. In addition, our studies, which need to be confirmed, suggest that at least on the basis of sequence analysis of a region of the L segment there are at least two distinct phylogenetic groups. These findings indicate that there may be significant sequence diversity and indeed that there may be distinct genetic subtypes within the LPHVs, which would be consistent with the known genetic diversity in related viruses. Studies on CCHFV have shown the greatest degree of sequence diversity of all known arboviruses and particularly in the M genome segment^19^,^20^. In CCHFV, sequence variation is considered to be primarily due to the error-prone nature of the vRNA polymerase. However there is evidence that genetic re-assortment can also occur. This is often seen in phylogenetic studies as an anomaly in the position of the M segment, whereas the S and L segments are located in their expected positions^28^. Further studies are required to determine the basis of genetic variation within the LHPVs.

Three viral isolates, designated 11SB17, 11SB19 and 11SB23 of LPHV were successfully obtained following cell culture of lung and liver tissues. Notably, all three belonged to the same major phylogenetic group based on the nucleotide sequences of the L segment as described above. The genomic organization of the three strains were typical of bunyaviruses having a tri-segmented genome. Nucleotide and amino acid sequences of the 11SB17 and 11SB19 strains had 99.97% homology and they were considered identical. However, comparison of the 11SB17 and 11SB23 strains demonstrated that while the L and N genes were identical the GPC gene in the M segment encoding the glycoprotein precursor proteins had only 68% nucleotide and 73% amino acid identity. In contrast, the untranslated regions in both termini of the M segment were highly conserved between the 11SB17 and 11SB23 strains (94% identity at the nucleotide level, Supplementary Figure 6). The GPC gene of CCHFV encodes a polyprotein, which encodes a mucin-like domain (MLD), GP38, the Gn protein, the NSm non-structural protein and the Gc protein. The MLD in the 11SB17 and 11SB23 strains exhibited low homology (29.1% at the amino acid level) with CCHFV. However, the calculated densities of potential O-glycosylated residues in this domain was conserved (30 of 102 and 35 of 95 residues; 31.3% and 37.2%, respectively). The amino acid sequences of the MLD are also known to vary among CCHFV strains^23^ and despite the aforementioned differences it is possible that the biological properties of MLD may well be maintained in the LPHVs. The Gn and Gc structural proteins of the LPHVs demonstrated similarities to those of CCHFV on BLAST search. However, while the MLD and the Gn and Gc regions were conserved, the inter domain regions were not, and indeed the LPHVs did not encode the non-structural NSm protein.

A previous study demonstrated that that the observed genetic differences in nairoviruses reflects the diversity of their predominant tick hosts and it was suggested that there could be co-evolution of the viruses and the latter^20^. Specifically this involved a phylogenetic analysis of a region of the L gene and could correlate nairovirus serogroups with their specific tick hosts. We carried out a similar analysis of the LHPVs and found that while they formed a distinct phylogroup they clustered closely with the Qalyub serogroup, which is known to be transmitted by soft tick, genus *Ornithodoros.* Notably our analysis showed that the genetic distance between the LPHVs and the Qalyub serogroup was similar to that between the Hughes and Dera Ghazi Khan serogroups thus supporting the view that the LPHVs can be considered to be an independent serotype. We examined all of the bats in our study for evidence of infestation by ticks but none could be demonstrated. Further studies are required to investigate this and to determine what role if any that ticks might have in the transmission of the LPHVs.

To characterize the clinical and pathogenic features of the LPHVs, viral isolates were used to infect adult C57BL/6J inbred mice. I.p. infection of the 11SB23 strain resulted in severe body weight loss, severe clinical symptoms and high mortality even following low-dose inocula. Significantly, high-dose challenge with the 11SB23 strain caused 100% mortality. In contrast, the 11SB17 strain only caused slight weight loss and no mortality even with high-dose challenge. Pathologically, 11SB23 infection was characterized by hepatic necrosis and small intestinal haemorrhage. All of 11SB23-infected mice, which were killed or moribund, exhibited the same gross pathological changes in the liver and intestine. Histopathological examination revealed hydropic and vacuolate degeneration and necrosis of hepatocytes and intestinal mucosal epithelial cells. Bleeding in the small intestine and exfoliation of the vacuolated mucosal epithelial cells were also observed. In addition, there was extensive necrosis of splenocytes. In contrast, no pathological changes were observed in 11SB17-infected mice. Biochemistry testing was consistent with severe liver injury and was associated with high vRNA loads in the liver and intestine. The observed high levels of LPHV replication in the liver was also consistent with the massive liver injury. In the 11SB23-infected mice, hypoglycemia was also present at 4 d.p.i. This could be due to starvation but possibly was also the result of acute liver failure. LPHV infection also resulted in severe leucopenia and thrombocytopenia in mice at 2 and 4 d.p.i., respectively. At these time points, viremia was observed following infection by both 11SB23 and 11SB17. However, the mean copy number of vRNA in 11SB17-infected mice was 10 times lower than that of 11SB23-infected mice at 2 d.p.i. At 4 d.p.i., the values in the 11SB23-infected mice had increased from that at 2 d.p.i. In contrast, the value of the 11SB17-infected mice at 4 d.p.i. was <10 times from 2 d.p.i. Overall the levels of vRNA appeared to reflect the severity of the clinical disease. These findings are similar to CCHFV infection in humans^29^, and also in Severe Fever with Thrombocytopenia Syndrome Virus infection^30^ and are considered important features of certain arboviral diseases.

To determine whether the differences in the pathogenicity of the two LPHV isolates could be related to possible differences in growth kinetics, we compared their replication rates in different cell lines including IFN-deficient Vero E6 cells, IFN competent HuH-7 cells and IFN competent Tb1Lu cells. The studies demonstrated that the growth kinetics were closely similar in all three cell lines, suggesting that this is probably not a significant factor. We also attempted to determine if the LPHVs contain the vOTU domain in the L protein and whether this might be related to the different pathogenic properties. Previous studies have shown that this domain can serve as a virulence factor and that this is involved in the modification of innate immune responses in several virus infections including CCHFV^21^. Our studies could demonstrate that the LPHVs do contain a vOTU domain and that this is most closely related to that in DUGV with 37% amino acid identity. However this domain was remarkably conserved with only one amino acid difference (R to K) being observed between the 11SB17 and 11SB23 isolates. In view of the fact that this change did not alter the basic property of the amino acid, it is felt that the vOTU domain may not contribute to the observed differences in the pathogenicity of the two isolates.

In conclusion, C57BL/6J mice were susceptible to LPHV infection and infection with one strain of LPHV resulted in a severe haemorrhagic gastroenteritis. The presentation of haemorrhage, pathological changes, blood cell counts and biochemical changes closely resemble those seen with CCHFV in humans^29^. In inbred mice, nairoviruses such as CCHFV, HAZV and DUGV have not been shown to cause disease. These viruses only cause lethal disease in suckling mice and type I interferon production or response-deficient mice^17^,^31^,^32^,^33^,^34^,^35^,^36^. Indeed, only immunodeficient and immature mice develop symptoms and succumb to infection with these nairoviruses. Notably, however, none of these are associated with haemorrhagic manifestations. Thus, the LPHV (11SB23 strain) infected C57BL/6J mice model offers a unique opportunity for the investigation of nairoviral haemorrhagic disease, and could provide important insights to that occurring in CCHFV infection. One additional advantage of this LHPV model is that animal experiments can be carried out in a BSL-3 facility, whereas experiments with other haemorrhagic fever-related viruses must be carried out in BSL-4 laboratories. Moreover studies of both the pathogenic and non pathogenic isolates of LPHV will potentially allow a better understanding of, and insights into, the molecular mechanisms involved in the development of disease.

## Methods

### Bat collection and ethical approval

Bat collection was performed with permission from the Zambia Wildlife Authority following guidelines contained in The Zambia Wildlife Act, 1998 (No.12 of 1998; http://www.parliament.gov.zm/dmdocuments/Wildlife%20Act%2012%201998.PDF). Debilitated bats were killed with ether prior to dissection. Animal specimens were transported from Zambia to Japan in accordance with the memorandum of understanding between the Research Center for Zoonosis Control, Hokkaido University and the School of Veterinary Medicine, University of Zambia. Animal experiments were performed in a Biosafety level 3 (BSL-3) laboratory at the Research Center for Zoonosis Control, Hokkaido University with approval from the Animal Care and Use Committee of Hokkaido University following Fundamental Guidelines for Proper Conduct of Animal Experiment and Related Activities in Academic Research Institutions under the jurisdiction of Ministry of Education, Culture, Sports, Science and Technology in Japan (approval number 12-0095).

### Sample collection and NGS analysis

Nine bats were collected in 2010, 30 in 2010, 20 in 2012 and were designated as 10SB01–09, 11SB01–30, and 12SB01–20, respectively. After euthanasia, the bats were dissected and lung, spleen, liver and kidney tissues were collected. Samples were stored at –80 °C prior to use. Samples from 10SB bats were used only for RNA extraction from spleen tissue. Tissues collected in 2011 and 2012 were individually homogenized in Dulbecco's minimal essential medium (DMEM) supplemented with 2% foetal bovine serum (FBS). Liver and kidney tissues from 11SB01–20 and 12SB01–20 and liver tissue from 11SB21–30 were homogenized. Liver and kidney homogenates from individual animals were mixed and all homogenates were used to inoculate Vero E6 cells. After 2 h incubation, the inoculum was replaced with fresh DMEM. In initial studies, cells were incubated for 14 days with one media change at 7 d.p.i. At 14 d.p.i., culture supernatants were harvested, centrifuged at 1,600*g* for 10 min, and stored. Supernatant aliquots (50 μl) from each year were prepared and designated as follows 11SB-lung, 11SB-mix (liver and kidney from 11SB01 to 11SB20), 11SB-liver (liver from 11SB21 to 11SB30) for 2011 samples, and 12SB-lung and 12SB-mix (liver and kidney from 12SB01 to 12SB20) for 2012 samples, respectively.

RNAs were extracted from supernatants using TRIzol LS reagent and a PureLink RNA Mini Kit (Invitrogen, Carlsbad, CA, USA), without DNase treatment. Double-stranded cDNA samples for next-generation sequencing were prepared using a cDNA synthesis kit (M-MLV version; Takara Bio, Ohtsu, Japan). To amplify cDNA libraries, random hexamer oligonucleotides with Tag sequences for PCR (5′-CGCTCTTCCGATCTNNNNNN-3′) were used as primers for first-strand cDNA synthesis according to previous studies^37^. Synthesized double-stranded cDNA samples were amplified using PCR with a Takara Ex-Taq polymerase (Takara Bio), the primer (5′-CGCTCTTCCGATCT-3′) and a reaction protocol of 2 min at 95 °C, 30 cycles of 15 s at 95 °C, 15 s at 40 °C, 30 s at 72 °C and then 5 min at 72 °C. Amplified samples were used in NGS analysis using a GS Junior sequencer (Roche, Basel, Switzerland) following the manufacturer’s instruction with nebulizing processes. The NGS data were deposited in the DDBJ Sequence Read Archive (DRA) under accession code DRA001134.

### Analysis of NGS data

Nucleotide sequence data from the NGS analyses were trimmed, and the Tag sequences were removed using CLC Genomics Workbench software version 5.0 (CLC bio, Aarhus, Denmark). After discarding short sequences (<100 bp), the fragments were analyzed using the BLASTN programme with default parameters. Viral sequences in the NCBI nt database were searched using the NCBI BLAST+ programme version 2.2.26 package^38^. Sequence fragments that had E values of <10^−3^ in BLAST search were selected and were analyzed using MEGAN software^39^ version 4.62.5 (http://ab.inf.uni-tuebingen.de/software/megan/). Lowest common ancestor analysis with MEGAN assigned sequence reads into 10 hierarchical levels, including kingdom, phylum, class, order, family, varieties, genus, species group, subspecies and species.

### Virus screening and species identification of bats

Total RNA samples were extracted from bat spleen tissues using the PureLing RNA Mini Kit after homogenization in TRIzol reagent. Spleen RNA samples from 10SB, 11SB and 12SB were screened for the newly identified nairovirus strains using one-step RT–PCR with a PrimeScript One-Step RT–PCR Kit Ver.2 (Takara Bio), primers designated for the L gene of the virus (5′-TGCATATCAGGAGACAATACAAAGTGG-3′ and 5′-AAAATGAATTTGATAACAGCWGGRGTTA-3′), with a reaction protocol of 30 min at 50 °C, 2 min at 95 °C, 45 cycles of 30 s at 95 °C, 30 s at 50 °C and 1 min at 72 °C and then 5 min at 72 °C.

Bat species were identified on the basis of *cytb* gene sequences. *cytb* fragments were amplified from spleen RNA using the one-step RT–PCR method with universal primers for bats (5′-CCATGAGGCCAAATATCCTTCTGAGG-3′ and 5′-TTGGCCAATGATAATGTAKGGRTGTTC-3′) and with a reaction protocol of 30 min at 50 °C, 2 min at 95 °C, 35 cycles of 30 s at 95 °C, 30 s at 50 °C and 1 min at 72 °C and then 5 min at 72 °C. Nucleotides were directly sequenced using the PCR primer or after cloning amplicons using a TA cloning Kit (Invitrogen). The 16 determined sequences of the L gene fragment from the newly identified nairoviruses and the sequences of *cytb* fragments from virus-infected bats were deposited in the DDBJ database under accession codes AB842072 to AB842087 and AB841413 to AB841428, respectively.

### Isolation of viruses and whole-genome analysis

Homogenized liver tissues positive for virus RNA in RT–PCR screening experiments were inoculated onto Vero E6 cells. Levels of LPHV RNAs were compared in the 0- and 6-day supernatants by using RT–PCR using the above screening primers. Increased levels of LPHV RNAs were detected in cultures inoculated with 11SB17, 11SB19 and 11SB23 tissue homogenates, and adenovirus DNA was detected in cultures with 12SB03 homogenates. Culture supernatants were stored as P0 virus samples, which were then propagated in Vero E6 cells (P1). Whole virus genome sequences were determined from P1 viruses using the NGS Roche GS Junior and previously described methods^16^. The terminal regions of each LPHV genome could not be analyzed with this NGS approach and were therefore amplified using an inverse PCR method. Virus RNA was self-ligated using thermostable single-stranded RNA ligase (Epicentre Biotechnologies, Madison, WI, USA) at 60 °C for 1 h and was amplified using a one-step RT–PCR from outside of the ligated termini. Primers were designed on the basis of the sequences obtained in NGS analyses and are listed in Supplementary Table 2. Amplification was performed using a reaction protocol of 30 min at 50 °C, 2 min at 95 °C, 45 cycles of 30 s at 95 °C, 30 s at 55 °C, and 1 min at 72 °C and then 5 min at 72 °C. RT–PCR products were purified using the MonoFas DNA purification kit I (GL sciences, Tokyo, Japan). The first PCR product was amplified using a nested PCR method with the primers listed in Supplementary Table 2 and with the reaction protocol of 2 min at 95 °C, 45 cycles of 20 s at 95 °C, 15 s at 55 °C and 30 s at 72 °C and then 5 min at 72 °C. Nucleotide sequences were determined directly using nested PCR primers. The terminus of the segment was based on the most conserved nairovirus terminal sequences. All experiments using unidentified or uncharacterized live viruses, including LPHVs, were performed in the BSL-3 laboratory at Research Center for Zoonosis Control, Hokkaido University. Complete nucleotide sequences of the LPHV genomes were deposited in the DDBJ database with the following accession codes: 11SB17 strain (L segment, AB842088; M segment, AB842089 and S segment, AB842090) and 11SB23 strain (L segment, AB842091; M segment, AB842092 and S segment, AB842093). Nucleotide sequences of the partial genome were deposited in the DDBJ database with the following accession codes: 11SB19 strain (AB842094, AB842095 and AB842096).

### Phylogenetic analysis

The obtained reads for virus genomes by NGS were assembled and analyzed using the CLC Genomics Workbench software version 5.0. Viral open reading frames, genes and translated amino acid sequences were deduced using GENETYX software ver. 10.1.0 (Genetyx Co., Tokyo, Japan). Homologies of sequences among bunyaviruses were calculated using CLC Genomics Workbench software. Amino acid sequences were aligned using the ClustalW algorithm and were phylogenetically analyzed using MEGA5 software^40^. The evolutionary histories of the family *Bunyaviridae* were inferred using the maximum likelihood method based on amino acid sequences of GPC protein. The relationship between serotype and genetic distance among nairoviruses was inferred using the maximum likelihood method based on amino acid sequences of partial L protein. Both the analyses were based on the JTT matrix-based model^41^. Initial trees for the heuristic search were obtained automatically by applying neighbour joining and BioNJ algorithms to a matrix of pairwise distances estimated using the JTT matrix-based model and then selecting the topology with the highest log-likelihood value. Signal peptides, transmembrane domains and O-glycosylation sites in amino acid sequences were predicted using Signal IP, TMHMM and NetOglyc programmes on the CBS server (http://www.cbs.dtu.dk/services/). Accession codes for the sequences are as follows: CCHFV (NC_005301, NC_005300 and NC_005302), DUGV (NC_004159, NC_004158 and NC_004157), ERVV (JF911697, JF911698 and JF911699), HAZV (DQ076419, DQ813514 and M86624), NSDV (HM991306, HQ286599 and HQ286602), Hantaan virus (NC_005222, NC_005219 and NC_005218), Seoul virus (GQ274942 and GQ274944), Heartland virus (JX005845 and JX005843), Rift Valley fever virus (NC_014396 and NC_014395), severe fever with thrombocytopenia syndrome virus (NC_018138 and NC_018137), Akabane virus (KC759123 and KC759124), Oropouche virus (NC_009895 and NC_009896) and tomato spotted wilt virus (NC_002050 and NC_002051). A data set of the partial L fragment has been previously reported^20^ (PopSet accession code 37550735).

### Clinical and pathological studies

C57BL/6J female mice obtained from the Jackson Laboratories were purchased from Hokudo Co. Ltd (Sapporo, Japan) and were infected at 8 weeks-of-age. Animal experiments were performed in a BSL-3 laboratory at the Research Center for Zoonosis Control, Hokkaido University with approval from the Animal Care and Use Committee of Hokkaido University following Fundamental Guidelines for Proper Conduct of Animal Experiment and Related Activities in Academic Research Institutions under the jurisdiction of the Ministry of Education, Culture, Sports, Science and Technology in Japan.

The titres of virus used in this study were defined in f.f.u.. Vero E6 cells were infected with LPHV and the infected and non-infected cells were cultivated in DMEM containing 1.25% methylcellulose and 2% FBS on a 48-well cultivation plate for 3 days. The cells were fixed with 10% formalin solution for 30 min and permeabilized by PBS containing 0.5% Saponin for 30 min. After blocking with PBS containing 0.1% BSA (Immuno-fluorescent assay buffer; IFA buffer) for 10 min, the Vero E6 cells were incubated with anti-LPHV mouse serum for 1 h. The mouse serum against LPHV were harvested from each LPHV-infected mouse at 21 d.p.i. and diluted to 1,000-fold by IFA buffer. After washing three times with IFA buffer, the cells were incubated with Alexa488-conjugated anti-mouse IgG (invitrogen) for 1 h. Fluorescent foci were counted under fluorescence microscopy. The staining procedure was performed at room temperature. Staining with the anti-LPHV mouse sera clearly demonstrated LPHV antigens in the cells (Supplementary Figs 7 and 8). The same lots of serum were used to measure f.f.u. LPHV 11SB17 and 11SB23 strains, which were passed twice in Vero E6 cells (P2 virus) were inoculated onto Vero E6 cells, at a 0.01 multiplicity of infection. At 3 d.p.i., the culture supernatants were harvested and centrifuged at 3,000 r.p.m. for 10 min. The supernatant was concentrated 10 times by ultracentrifugation at 100,000*g* for 2 h (P3 virus). Each P3 virus contained 2.5 × 10^7^ f.f.u. ml^−1^ in PBS.

For mouse infection experiments, 5 × 10^6^ f.f.u. virus and 5 × 10^5^ f.f.u. virus was i.p injected into C57BL/6J mice as high-dose and low-dose challenges, respectively. PBS was used as a NC. The injected mice were observed and weighed every day. Upon developing symptoms of severe disease, including a >25% decrease in body weight and hunched posture without movement, the mice were anesthetized and blood samples were harvested from the heart and other tissues. Other mice that were also bled from the heart and tissue specimens were harvested under anaesthesia at the indicated time point or the specified 21 d.p.i. end point. Portions of harvested tissue specimens were fixed using phosphate-buffered 10% formalin, and were embedded in paraffin and stained with haematoxylin–eosin. Haematology and blood biochemistry were tested using VetScan HM II and VetScan VS2 with Multi-Roter II VCDP cartridge (Abaxis, Union City, CA, USA), respectively.

Total RNA was prepared from 50 μl of the collected blood in the representative three individuals using a PureLink RNA Mini Kit and TRIzol reagent. Quantities of LPHV virus RNA were measured using qRT–PCR with a Brilliant III Ultra-Fast qRT–PCR Master Mix (Agilent Technologies, Santa Clara, CA, USA), and specific primers for the L gene (5′-TCCTAATCACACCTTTGCCTCT-3' and 5′-TCCTATCCTTCCTTGCTCTCTC-3′). The qRT–PCR reaction protocol was 10 min at 50 °C, 3 min at 95 °C and then 40 cycles of 5 s at 95 °C and 10 s at 60 °C. Length of the PCR product is 105 bp. Nucleotide sequence of the target region was identical between 11SB17 and 11SB23 strains and the corresponding amplicon was cloned into pCR4-Blunt vector (Invitrogen). The cloned plasmid was deduced the copy number and used for the standard of the measurement. QRT–PCR was performed using a StepOne plus qPCR system (Applied Biosystems), and the data were analyzed using StepOne software verson 2.3.

### Titration of IgG antibody in LPHV-infected mice

To prepare antigens for ELISA, the LPHV 11SB17 and 11SB23 isolates were used to infect Vero E6 cells. Non-infected Vero E6 cells were used as a NC. After 2 days of cultivation, cells were lysed with lysis buffer (100 mM Tris-HCl (pH 7.5), 150 mM NaCl, 1 mM EDTA, 1 mM EGTA, 1% Triton-X, 0.5% sodium deoxycholate and 1 mM PMSF). Lysed cells were diluted to a concentration of 10 μg ml^−1^ protein and employed as ELISA antigen. Ninety-six-well plates were coated with 25 μl of the antigen protein solution overnight at 4 °C. After removal of the protein solution, the coated wells were blocked with 100 μl of 5% skim milk in PBS for 1 h at 37 °C. After discarding the blocking solution and washing once with PBS, 100 μl of 1,000-fold or 10,000-fold diluted mouse serum in 1% skim milk in PBS was added to each well. Following incubation for 1 h at 37 °C, the wells were washed once with 150 μl of PBS containing 0.01% Tween 20. Thereafter, 100 μl of 20,000-fold diluted horseradish peroxidase (HRP)-conjugated anti-mouse IgG (Biosource, Camarillo, CA, USA) with 1% skim milk in PBS was added. After incubation for 1 h at 37 °C, the wells were washed four times with 150 μl of PBS containing 0.01% Tween 20. To measure HRP activity, TMB Super Sensitive One Component HRP Microwell Substrate and 450 nm Liquid Stop Solution for TMB (SurModics, Eden Prairie, MN) were used according the manufacturer’s instructions. Values of absorbance were measured at 450 nm in a Model 680 microplate reader (Bio-Rad, Hercules, CA, USA).

## Author contributions

A.I. conceived the research. A.I., Y.O., M.S., L.M., B.M.H, A.S.M. and H.S. collected the samples. A.I., K.U., Y.O., T.U. and H.S. performed the experiments. A.I., T.U., K.I., W.W.H. and H.S. analyzed the data. A.I., W.W.H. and H.S. wrote or revised the manuscript.

## Additional information

**How to cite this article:** Ishii, A. *et al.* A nairovirus isolated from African bats causes haemorrhagic gastroenteritis and severe hepatic disease in mice. *Nat. Commun.* 5:5651 doi: 10.1038/ncomms6651 (2014).

**Accession codes:** The NGS sequences have been deposited in the GenBank/EMBL/DDBJ databases under accession code DRA001134. The sequences for partial cytochrome b gene in bat, partial L gene of LPHV, and genome of LPHV have been deposited in the GenBank/EMBL/DDBJ databases under accession codes AB841413 to AB841428, AB842072 to AB842087, and AB842088 to AB842096, respectively.

## Supplementary Material

Supplementary Information Supplementary Figures 1-8 and Supplementary Tables 1-2

## Figures and Tables

**Table 1:** Pairwise comparisons of nucleotide and amino acid sequences among bunyaviruses.

**Table 2:** Average values of blood biochemistry and haematology.

	**ALT**	**ALP**	**TBIL**	**GLU**	**WBC**	**PLT**	**RBC**
*2 d.p.i.*							
NC	30.7	90.7	0.29	288.7	3.29	670.3	9.6
11SB17	290.1	67.4	0.29	248.4	1.18	323.1	9.8
11SB23	1,849.6	100.4	0.33	177.4	1.00	256.7	10.3
							
*4 d.p.i.*							
NC	30.0	76.3	0.30	287.6	4.19	698.6	9.5
11SB17	478.7	87.0	0.29	228.0	2.93	138.4	9.6
11SB23	>2,000	377.3	1.70	60.9	0.55	17.4	10.1

ALP, alkaline phosphatase (U L^−1^); ALT, alanine aminotransferase (U L^−1^); GLU, glucose concentrations (mg per 10^−1^ l); PLT, platelet (10^3^ per μl); RBC, red blood cells (10^6^ per μl); TBIL, total bilirubin (mg per 10^−1^ l); WBC, white blood cells (10^3^ per μl).
