# Supplementary material for: A nairovirus isolated from African bats causes haemorrhagic gastroenteritis and severe hepatic disease in mice
Source: Nat Commun. 2014 Dec 2;5:5651. doi: 10.1038/ncomms6651 (PMC4268697; doi:10.1038/ncomms6651)
Supplement: Supplementary Information — Supplementary Figures 1-8 and Supplementary Tables 1-2 [file ncomms6651-s1.pdf]

Supplementary Figure 1 - Ishii, A. et al

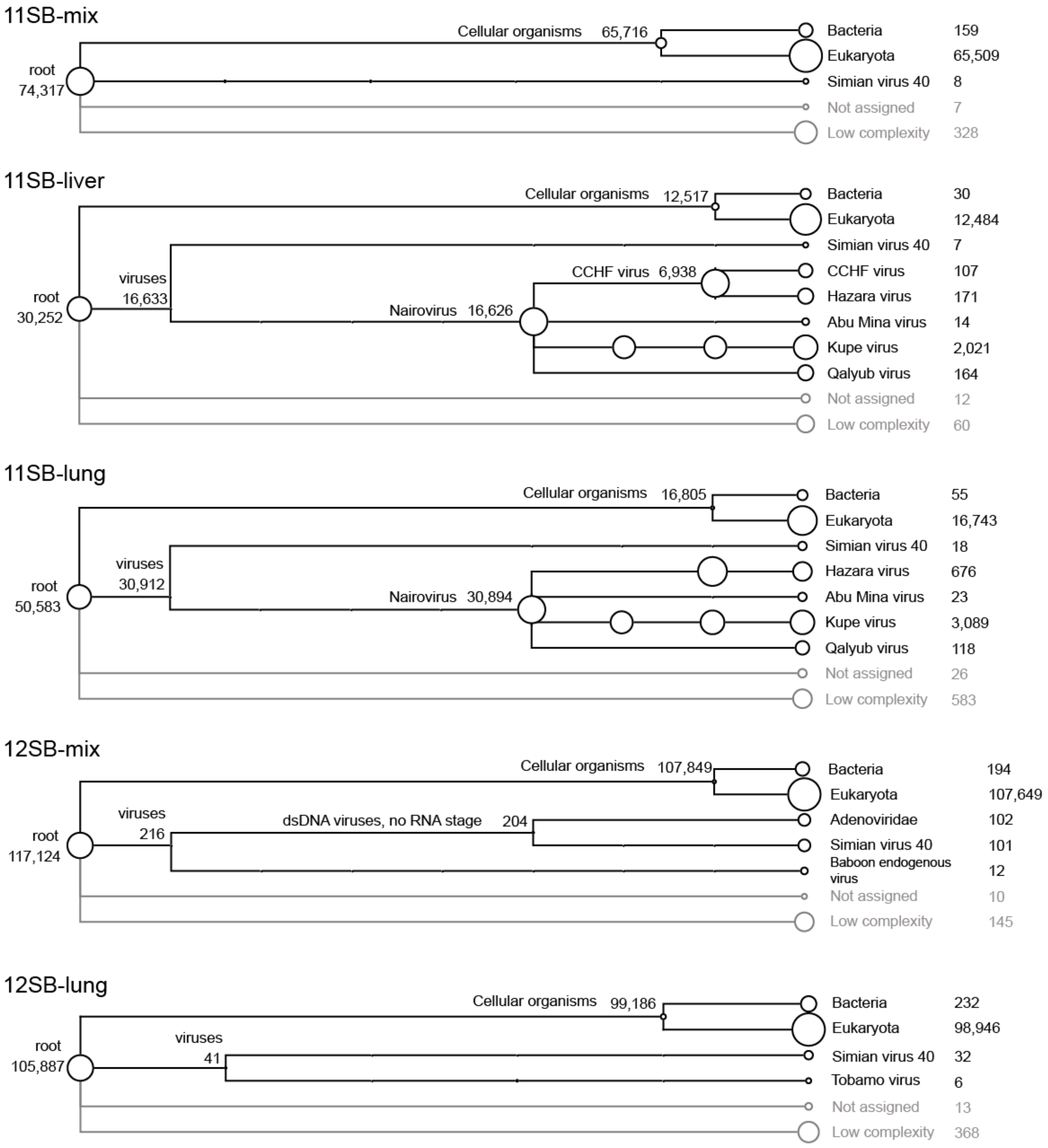

Supplementary Figure 1. BLAST results summarized using MEGAN software.

BLAST analyses of the reads obtained by NGS were summarized on the evolution tree for all living organisms using MEGAN software. Numbers of annotated reads are indicated beside the nodes.

Supplementary Figure 2 - Ishii, A. et al

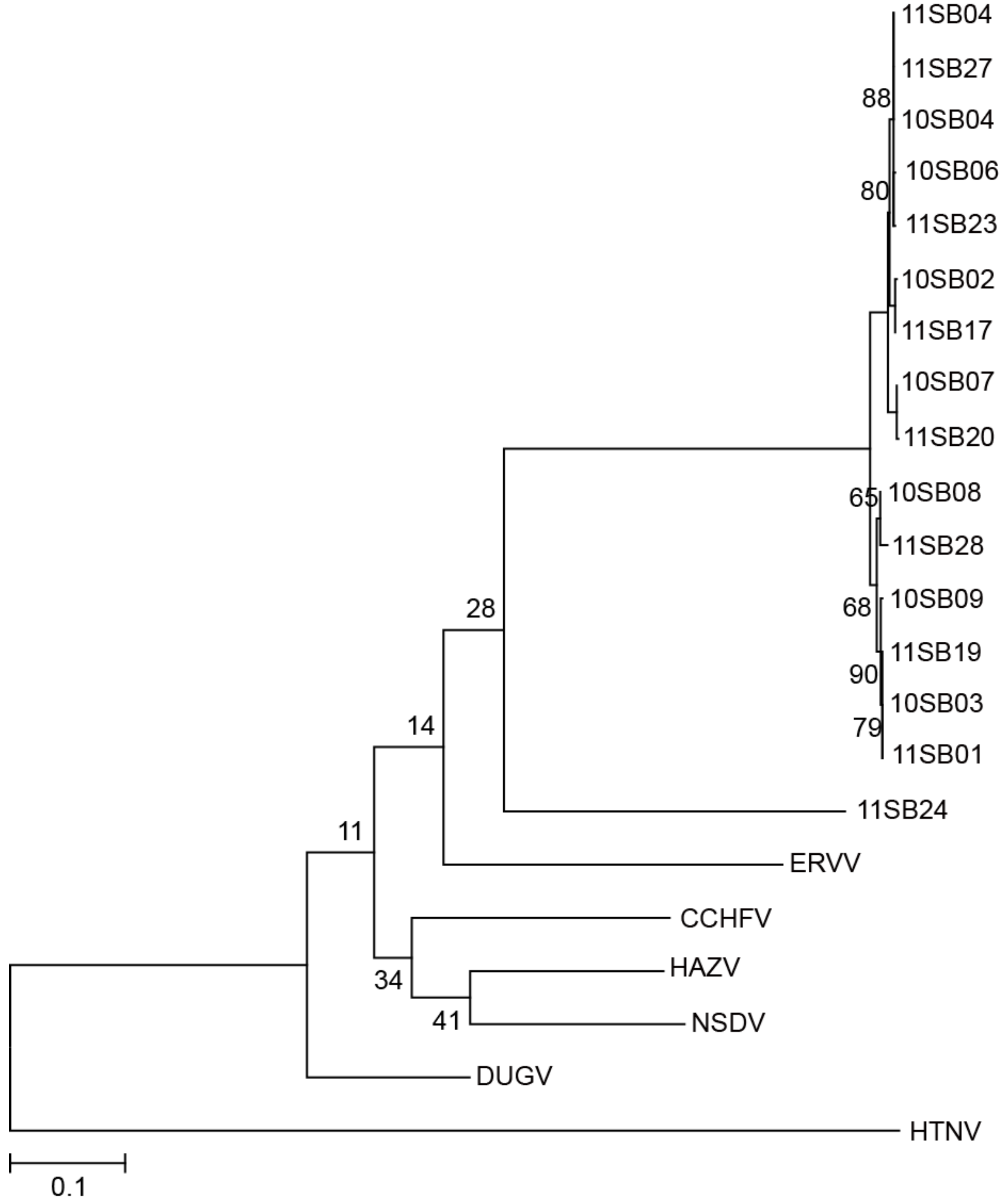

Supplementary Figure 2. Phylogenetic analysis of nairoviruses including the novel virus (LHPV).

Nucleotide sequences of L gene fragments were phylogenetically analyzed, and the tree with the highest log-likelihood value (−5295.0992) was shown. The percentages of trees in which the associated taxa are clustered are shown beside the branches. Initial trees for the

heuristic search were obtained automatically by applying neighbor-joining and BioNJ algorithms to a matrix of pairwise distances that were estimated using the maximum composite likelihood approach and then selecting topologies with superior log-likelihood values. The tree is drawn to scale, with branch lengths indicating the number of substitutions per site. The analysis involved 22 nucleotide sequences. Codon positions included were 1st + 2nd + 3rd + noncoding. All positions containing gaps and missing data were eliminated. There were a total of 635 positions in the final dataset. HTNV was defined as the outgroup. Leopards Hill virus (LPHV) strains are indicated by sample IDs: CCHFV, Crimean-Congo hemorrhagic fever virus; DUGV, Dugbe virus; ERVV, Erve virus; HAZV, Hazara virus; NSDV, Nairobi sheep disease virus; and HTNV, Hantaan virus.

Supplementary Figure 3 - Ishii, A. et al

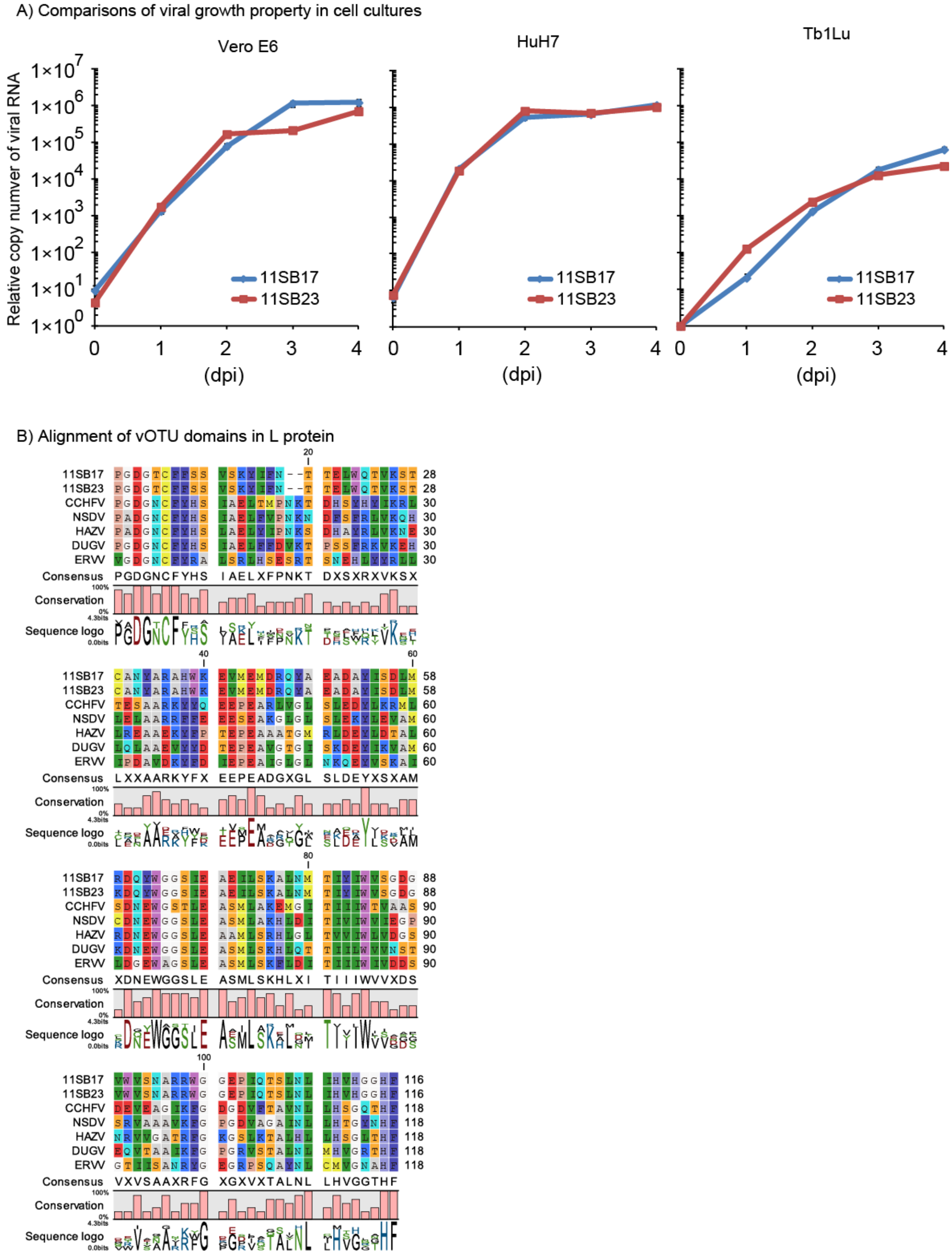

Supplementary Figure 3. Comparisons of growth property and vOTU-domain between 11SB17 and 11SB23. (A) LPHVs infected to Vero E6 cells, HuH-7 cells (human

hepato cellular carcinoma cells), and Tb1Lu cells (bat epitherial cell derived from free-tailed bat, Tadarida brasiliensis) with 0.01 moi and 50 ml of culture supernatant was sampled at 0, 1, 2, 3, and 4 days post infection (dpi). RNA was extracted from the supernatant and relative copy number of viral RNA was measured by one-step qRT-PCR. (B) Deduced amino acid sequences of viral Ovarian Tumor (vOTU)-domain in LPHVs were aligned with those of other nairoviruses.

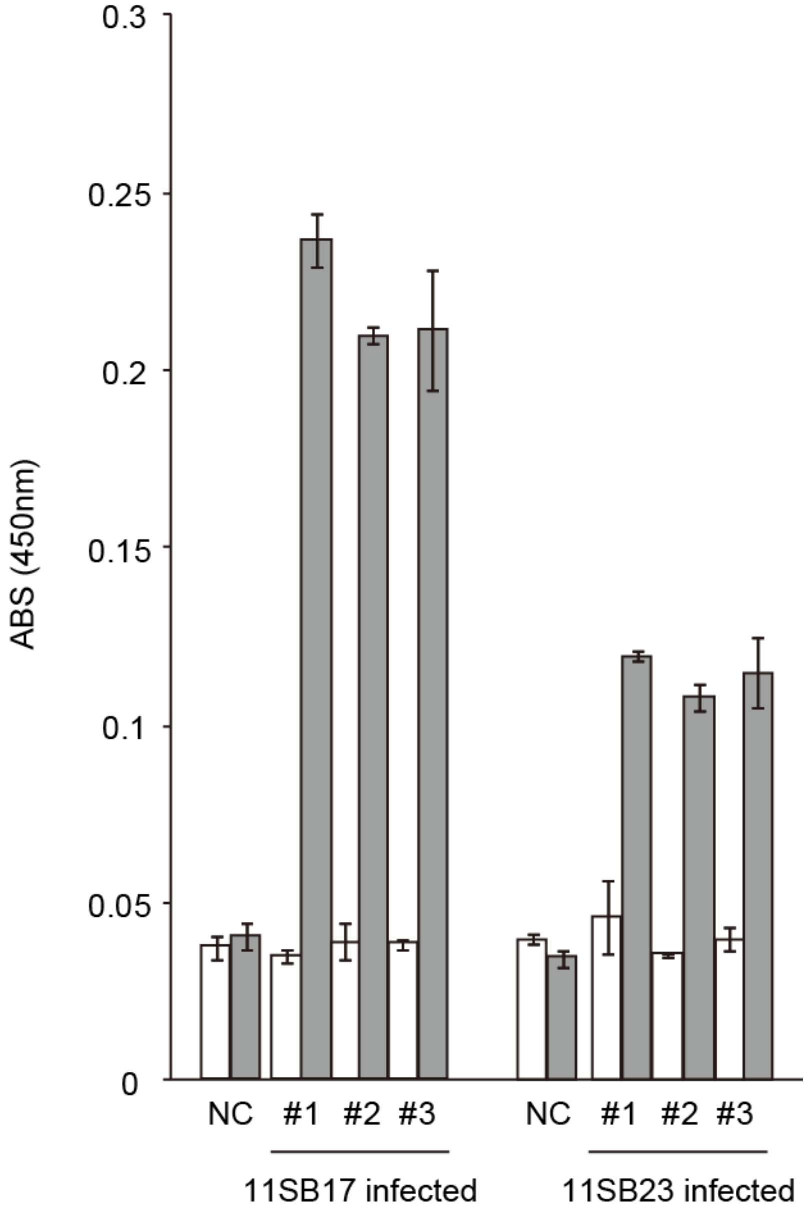

Supplementary Figure 4. Titration of IgG antibody for LPHV in infected mice.

Titer of IgG antibody for LPHV in infected mice were measured by ELISA. Sera were harvested from non-infected (NC), and 3 survived individuals of LPHV 11SB17-infected and 11SB23-infected mice at 21 dpi. Protein solutions from Vero E6 cells (open) and LPHV-infected Vero E6 cells (gray) were usedfor antigens.

Supplementary Figure 5 - Ishii, A. et al

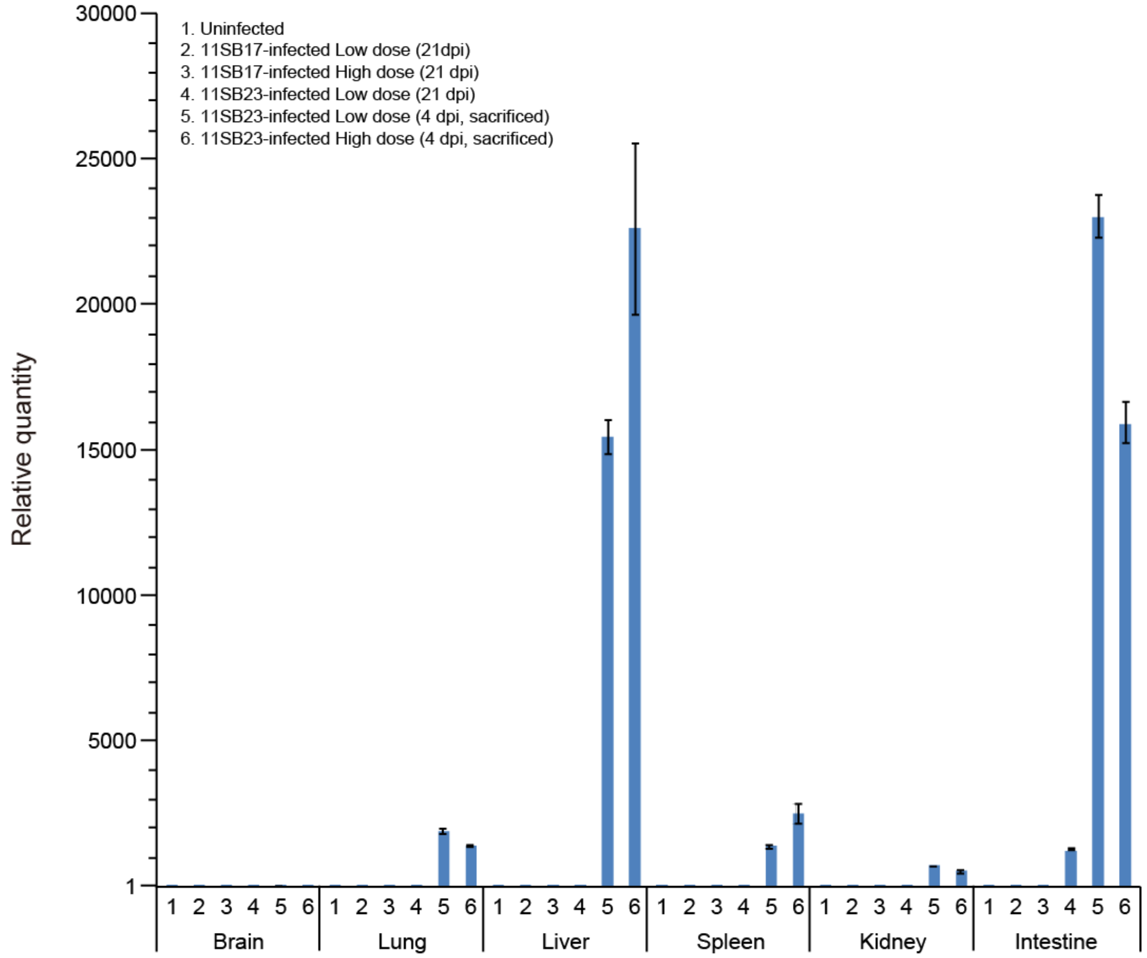

Supplementary Figure 5. Quantitative RT-PCR for virus RNA in mouse tissues.
Total RNA was prepared from the harvested mouse tissues using a PureLink RNA Mini Kit and TRIzol reagent. Quantities of LPHV virus RNA in 100 ng of RNA sample were measured using quantitative RT-PCR (qRT-PCR) with a Brilliant III Ultra-Fast qRT-PCR Master Mix (Agilent Technologies, Santa Clara, CA), and specific primers for the L gene (5'-TCCTAATCACACCTTTGCCTCT-3' and 5'-TCCTATCCTTCCTTGCTCTCTC-3'). The qRT-PCR reaction protocol was 10 min at 50°C, 3 min at 95°C, and then 40 cycles of 5 s at 95°C and 10 s at 60°C. Length of the PCR product is 105 bp. Nucleotide sequence of the target region was identical between 11SB17 and 11SB23 strains. Relative quantity for uninfected tissue was shown. qRT-PCR was performed using a StepOne plus qPCR system (Applied Biosystems), and the data were analyzed using StepOne software ver2.3. Each measurement was performed in triplicate, and data are expressed as mean ratio ± SD.

Supplementary Figure 6 - Ishii, A. et al

L segment

```
LPHV 11SB17   TCTCAAAGAAAGTAATCCCCCATATCCCAAATCATCAACAGGCCATG - AGCAGTAATGATTCGGGTATGGGGGAACAATATCTTTGAGA
LPHV 11SB23   TCTCAAAGAAAGTAATCCCCCATATCCCAAATCATCAACAGGCCATG - AGCAGTAATGATTCGGGTATGGGGGAACAATATCTTTGAGA
CCHF virus    TCTCAAAGATATCAATCCCCCCGTTACCCACGTTAACACAGAGAGCT - CTGTGTTAACGTGGGTAACGGGGGGATTGATATCTTTGAGA
Dugbe virus   TCTCAAAGACATCAATCCCCCTTTTCCCCAAAACTTAAACATGGACT - TGTTTAAGTTTTGGGGAAAAGGGGGATTGATGTCTTTGAGA
Erve virus    TCTCAAAGAAAGCAATCCCCCCAACTCACTACTACAAAATGGATGCT - CATTTTGTAGTAGTGAGTTGGGGGGATTGCTTTCTTTGAGA
Hazara virus  TCTCAAAGACATCATCCCCCTTATCCCCAAGTTAACATGGACTTTCT - TCCATGTTAACTTGGGGATAAGGGGGATGATGTCTTTGAGA
NSD virus     TCTCAAAGATATCAATCCCCCCGTTACCCCAGAGTTGCAAGCATGGA - CTTGCAACTCTGGGGTAACGGGGGGATTGATATCTTTGAGA
```

M segment

```
LPHV 11SB17   TCTCAAAGAAAGACTTGCGGCTACCCGTAATTCTTGAGAGATG - GAACATCACATACAGGGTATGCCGCCACTATATCTTTGAGA
LPHV 11SB23   TCTCAAAGAAAGACTTGCGGCTACCCGTAAACTTTGAGAGATG - AGAAACCTTTAATAGGGTATGCCGCCACTATATCTTTGAGA
CCHF virus    TCTCAAAGAAATACTTGCGGCACGTCAGTACGTAAGTGTTAAC - TAACACTTACGTACTGACGTGCCGCAAGTATTTCTTTGAGA
Dugbe virus   TCTCAAAGACATACCTGCGGCACATACAAGTGTTAAAGGTGTT - CACCTTTAACACTTGTATGTGCCGCAGGTATGTCTTTGAGA
Erve virus    TCTCAAAGAAAGACTAGCGGCAAACTCGTCTTACTTTGAGATG - TCTCAAAGTAAGACGAGTTTGCCGCTAGTCTTTCTTTGAGA
Hazara virus  TCTCAAAGACAGACTTGCGGCACACACAAAAGGAAACTCCAGT - TGGAGTTTCCTTTTGTGTGTGCCGCAAGTCTGTCTTTGAGA
NSD virus     TCTCAAGAGAGCAATCAAGATGGCATTAGTGGCAAAGGGTTTA - AACCCTTTGCCACTAATGCCATCTTGATTGCTCTCTTGAGA
```

S segment

```
LPHV 11SB17   TCTCAAAGAAAGACGTGCCGCTTACCCGTAAGCAAACATG - GAGTGGAATAGTTCGGGTATGCGGCAACATTATCTTTGAGA
LPHV 11SB23   TCTCAAAGAAAGACGTGCCGCTTACCCGTAAGCAAACATG - AAGTGGAATAGTTCGGGTATGCGGCAACATTATCTTTGAGA
CCHF virus    TCTCAAAGAAACACGTGCCGCTTACGCCCACAGTGTTCTC - AGAGAACACTGTGGGCGTAAGCGGCACGTGTTTCTTTGAGA
Dugbe virus   TCTCAAAGACAAACGTGCCGCAGTCGCCCGATTGTACTTT - CAAAGTACAATCGGGCGACTGCGGCACGTTTGTCTTTGAGA
Erve virus    TCTCAAAGAAAGTTGTGCTGTTACTGTTTCACACTTTCTT - AAAGAAAGTGTGAAACAGTAACAGCACAACTTTCTTTGAGA
Hazara virus  TCTCAAAGACAAACATGCCGCAGACGCCCCACGTTTTCAT - GATGAAAACGTGGGGCGTCTGCGGCATGTTTGTCTTTGAGA
NSD virus     TCTCAAAGACACACGTGCCGCTTTCGCCCGAATTGTCTTT - CAAAGACAATTCGGGCGAAAGCGGCACGTGTGTCTTTGAGA
```

Supplementary Figure 6. Alignment of nucleotide sequences of genome termini.

Nucleotide sequences of termini of each genome segment were aligned. Conserved terminal sequences were underlined.

Supplementary Figure 7 - Ishii, A. et al

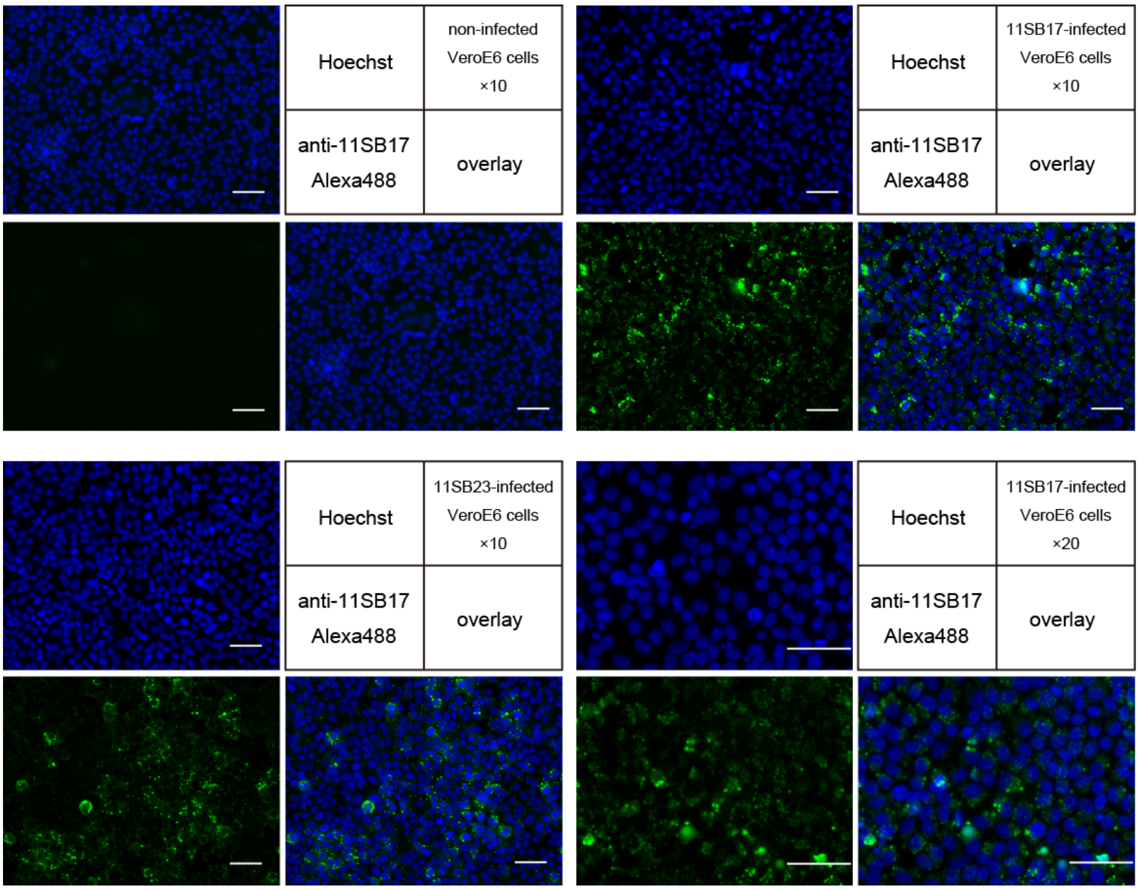

Supplementary Figure 7. Immuno-fluorescent stain of LPHV infected Vero E6 cells with serum from 11SB17 strain-infected mouse.

The infected virus strain, used serum for staining, and magnification of objective lens for observation were indicated in upper right panel. The same lot of serum was used for titration of LPHV 11SB17 strain for following mouse infection study. Scale bars (100 mm) are shown in the lower right side of the figures.

Supplementary Figure 8 - Ishii, A. et al

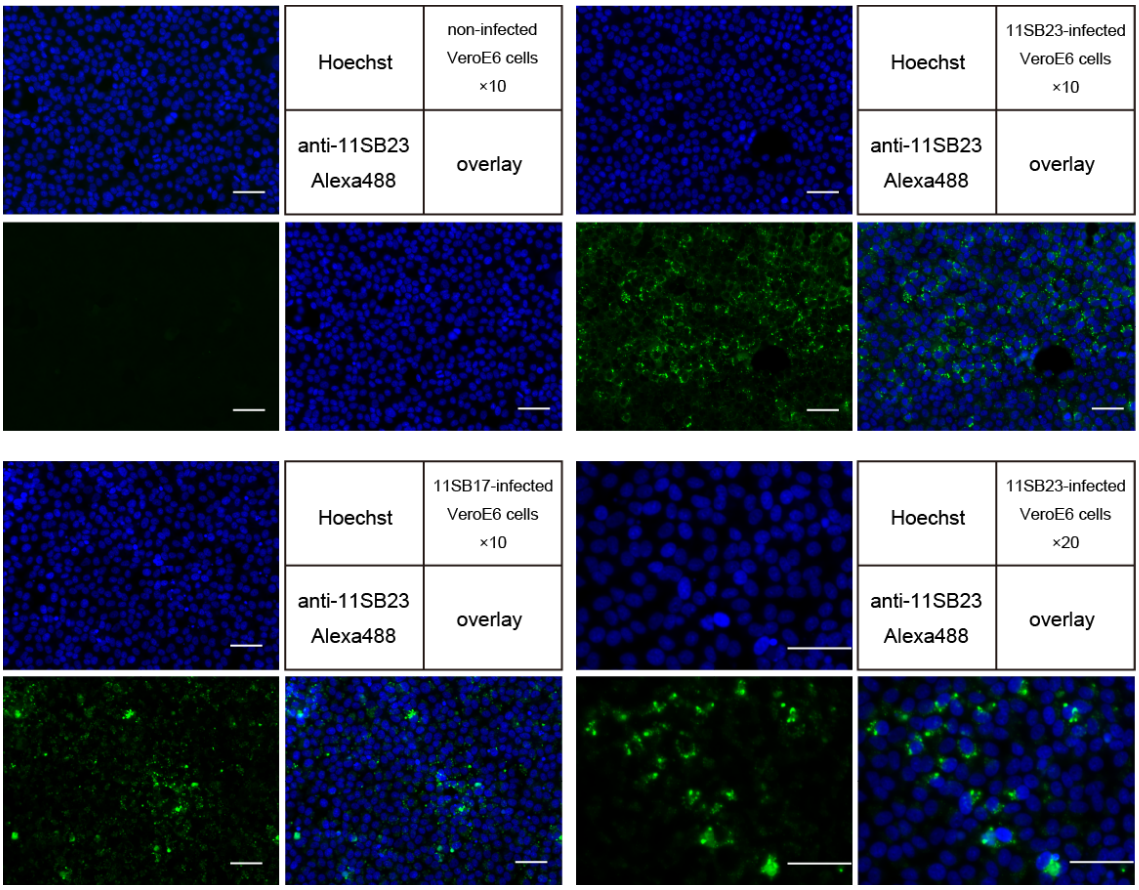

Supplementary Figure 8. Immuno-fluorescent stain of LPHV infected Vero E6 cells with serumfrom 11SB23 strain-infected mouse.

The infected virus strain, used serum for staining, and magnification of objective lens for observation were indicated in upper right panel. The same lot of serum was used for titration of LPHV 11SB23 strain for following mouse infection study. Scale bars (100 mm) are shown in the lower right side of the figures.

Supplementary Table 1. Percent identities of nucleotide and amino acid sequences.

strain	segment	length (bases)	gene	length (bases)
**11SB17**	L	12,037	L	11,904
	M	4,571	GPC	4,263
	S	1,760	N	1,545
**11SB19**	L	ND	L	11,904
	M	ND	GPC	4,263
	S	ND	N	1,545
**11SB23**	L	12,037	L	11,904
	M	4,504	GPC	4,233
	S	1,762	N	1,545

ND; not determined

Supplementary Table 2. Primers for terminal sequencing

**1st RT-PCR**			
**11SB17**			
	**L**	TCAGCAGAACAAGTTACCTATCTAGATCTC	TCTCCATCACTTCTTTCCAGTGTGCTC
	**M**	GCAGTATGCACTTTAATTAAGCTCAAACTC	TGAGTTGTGTCCATGCCCTTAGAAGTTG
	**S**	TTGAACTCCTCAAACCAGTTGTTGAGG	ACTCTCCCAGTGGCTCTTCGAACTG
**11SB23**			
	**L**	TCAGCAGAACAAGTTACCTATCTAGATCTC	TCTCCATCACTTCTTTCCAGTGTGCTC
	**M**	TCCCTGTGTAATAGCCTCAACTGTGTC	TGTTGCTTCAATCTACTAGTCATTCTAGC
	**S**	TTGAACTCCTCAAACCAGTTGTTGAGG	ACTCTCCCAGTGGCTCTTCGAACTG
**nested PCR**			
**11SB17**			
	**L**	AGATCTCTTCGAGGATTCACCGTCAGCAG	AGCATAGTTGGCACAGGTGCTTTTGACTG
	**M**	TAGATAATTTGAAGACGCAAGACATCCAG	TCTGTAGATCCAGTTGTATTACTGACAAC
	**S**	TCAAATCCTTAAAGTTCAGCTCAGACATG	ATGGAGATGCAAGGTGAATGAGAACAGC
**11SB23**			
	**L**	AGATCTCTTCGAGGATTCACCGTCAACAG	AGCATAGTTGGCACAGGTGCTTTTGACTG
	**M**	AGATCTCGACGTATTAGTCTCTCCCTGTG	TGGTAAGAAGAAGGAGTAAGTAGGCACC
	**S**	TCAAATCCTTAAAGTTCAGCTCAGACATG	ATGGAGATGCAAGGTGAATGAGAACAGC

Nucleotide sequences of primers used in inverse RT-PCR and nested PCR experiments.
